# Impact of different surgical procedures on survival outcomes of patients with adenocarcinoma of pancreatic neck

**DOI:** 10.1371/journal.pone.0217427

**Published:** 2019-05-24

**Authors:** Zhenjiang Zheng, Chunlu Tan, Yonghua Chen, Jie Ping, Mojin Wang

**Affiliations:** 1 Department of Pancreatic Surgery, West China Hospital, Sichuan University, Chengdu, Sichuan Province, China; 2 Division of Epidemiology, Vanderbilt University Medical Center, Nashville, TN, United States of America; 3 Department of Gastrointestinal Surgery, Institute of Digestive Surgery and State Key Laboratory of Biotherapy, West China Hospital, Sichuan University, Chengdu, Sichuan Province, China; VA Boston Healthcare System, Harvard Medical School (Brigham and Women's Hospital), UNITED STATES

## Abstract

**Background:**

The only curative treatment for pancreatic adenocarcinoma is radical surgical resection. Because of the special anatomic features of pancreatic neck, the selection of optimal surgical procedure for treatment of adenocarcinoma of pancreatic neck has always been a dilemma for surgeons. In this paper, we aim to investigate whether different surgical procedures can affect prognosis in the patient with adenocarcinoma of pancreatic neck.

**Methods:**

We used the surveillance, epidemiology, and end results database to review patients with adenocarcinoma of pancreatic neck diagnosed between 1998 and 2015. We calculated overall survival (OS) and cancer-specific survival (CSS) of these patients using Kaplan-Meier analysis and Cox regression model.

**Results:**

Overall, 1443 patients were included in the study, with 12.5% treated with surgical resection. Among them, 30 (18.8%) patients underwent distal pancreatectomy (DP), 105 (65.6%) patients underwent pancreatoduodenectomy (PD), and 25 (15.6%) patients underwent total pancreatectomy (TP). Patients underwent DP were older than these underwent TP (70.5±10.7 vs. 62.2±14.1, *P* = 0.027). Patients underwent TP had higher percentages of nodal metastasis (N1 stage) than these underwent DP (68.0% vs. 34.5%, *P* = 0.014). The surgical procedures did not significantly affect either OS times (*P* = 0.924) or CSS times (*P* = 0.786) in Kaplan-Meier analysis, even if in any subgroup of AJCC stage. The multivariate Cox regression model showed that types of surgery were not associated with OS and CSS. Higher tumor grade and AJCC stage are independent prognostic factors for OS and CSS. No radiotherapy was associated with a worse CSS (HR 1.610, 95% CI 1.016–2.554, *P* = 0.043).

**Conclusion:**

Different surgical procedures did not affect prognosis in the patients with adenocarcinoma of pancreatic neck. TP should be performed in carefully selective patients in high-volume pancreatic centers.

## Introduction

Pancreatic adenocarcinoma is one of most lethal disease with 8% overall 5-year survival.[1] The only curative treatment for pancreatic adenocarcinoma is radical surgical resection. Conventional surgical procedures for pancreatic adenocarcinoma are basically represented by pancreatoduodenectomy (PD) and distal pancreatectomy (DP), according to the tumor’s location.[2] Advances in surgical skills have allowed for evolution in pancreatic adenocarcinoma surgery. Thus, total pancreatectomy (TP) has become an alternative surgical procedure in high-volume pancreatic centers to achieve complete tumor resection with negative margins.[3] Pancreatic neck located in a short segment (approximately 2 cm) between pancreatic head and body, anterior to the portal vein (PV), on the left side of the gastroduodenal artery (GDA), and below the common hepatic artery (CHA).[4] These anatomic features resulted in different clinicopathologic characteristics of pancreatic neck cancer, as compared to cancer located in the head or in the body and/or tail of the pancreas.[4] For the treatment of benign diseases or low-grade malignancies in pancreatic neck, central pancreatectomy is appropriate, to preserve more pancreatic parenchyma and function.[5,6] However, this technique is improper in the setting of invasive tumor as parenchyma-sparing may lead to tumor lesion residual. Because of the special anatomic features of pancreatic neck, the selection of optimal surgical procedure for invasive tumor has always been a dilemma for surgeons. Few discussions on pancreatic adenocarcinoma has been focused on the pancreatic neck, due to these cases are often classified as pancreatic head or body cancer.[4] Furthermore, no studies to date have compared the impact of different surgical procedures on survival of adenocarcinoma of pancreatic neck.

In this study, we used the data from the Surveillance, Epidemiology, and End Results (SEER) database to investigate the impact of different types of surgery on the overall survival (OS) and cancer-specific survival (CSS) in patients with adenocarcinoma of pancreatic neck.

## Materials and methods

### Data source

Data were obtained from the SEER. The SEER program collects cancer incidence and survival from 18 population-based registries covering approximately 34.6% of the United States population (http://seer.cancer.gov/about/overview.html). This version of SEER database we used had been released April 2018 (November 2017 submission). The SEER registry provides information on demographics, tumor characteristics, treatment characteristics and survival. The permission was obtained to access the research data files (reference number 10457-Nov2017).

### Patient selection

All patients with a diagnosis of adenocarcinoma of pancreatic neck (age of diagnosis >18 years) from 1998 to 2015 were included in the study according to the International Classification of Disease for Oncology, third edition (ICD-O-3), site codes C25.7 (other specified parts of pancreas, e.g., neck) in combination with appropriate histology codes (8140 and 8500). For subgroup survival analysis of only patients underwent surgical resection, patients who had metastatic disease and those with unclear extent of surgery were excluded from analysis.

### Data analysis

Patients in the study cohort was divided into surgery group and no surgery group. For patients underwent surgical resection, data analysis was subdivided by types of surgery: DP group (code 30), PD group (codes 35–37, and 70), and TP group (codes 40 and 60). Tumor stages were classified according to the 7th edition of American Joint Committee on Cancer (AJCC) staging system. Tumor histological grade was categorized as well differentiated, moderately differentiated, poorly differentiated, and undifferentiated. OS and CSS were defined as time from diagnosis to death (all causes) and death due to cancer, respectively.

### Statistical analysis

All statistical analyses were performed with standard statistical programs (SPSS version 22.0; IBM-SPSS, Chicago, IL). We evaluated statistical differences using Student's t-test for continuous variables and Chi-square test and Fisher exact test for categorical variables. OS and CSS rates were calculated by Kaplan–Meier method, and differences were examined by log-rank test. The effects of demographic, tumor, and treatment characteristics on survival were analysed by univariate and multivariate Cox regression analysis. The resulting hazard ratios (HR) with its 95% confidence intervals (CI) were presented. All *P* values were 2-sided, and *P* <0.05 were considered as statistically significant.

## Results

### Analysis of all patients with adenocarcinoma of pancreatic neck

**Fig 1:**
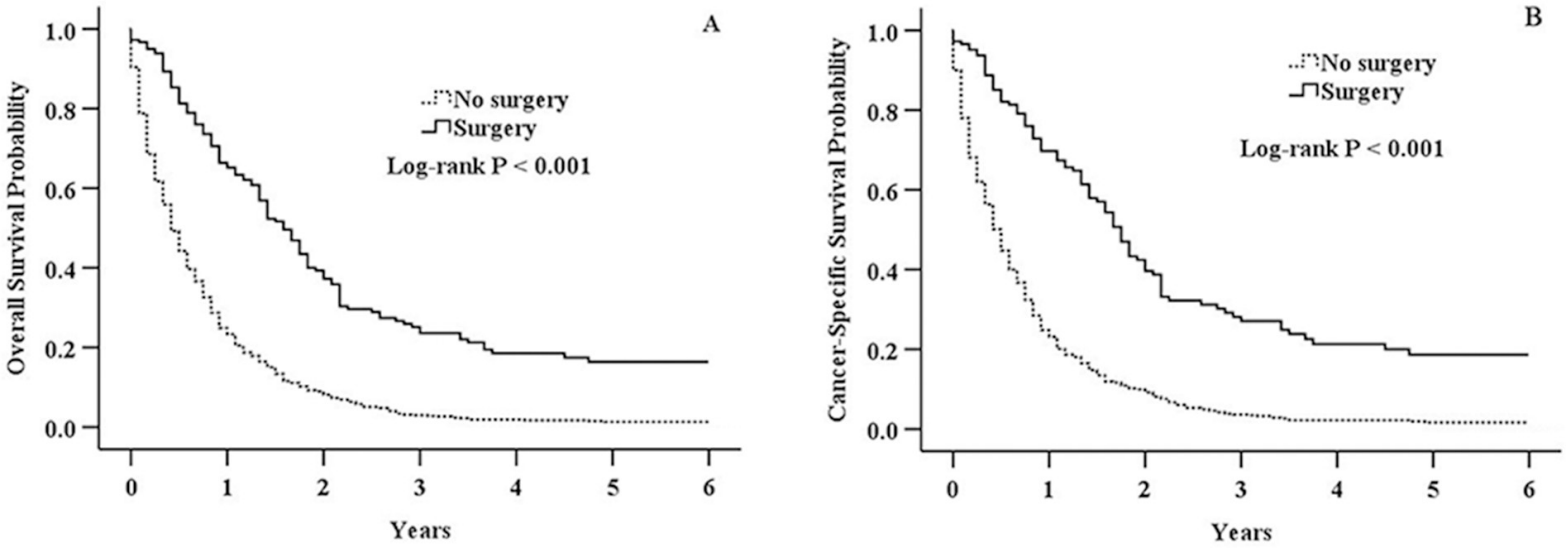
Kaplan-Meier survival analysis of all patients with adenocarcinoma of pancreatic neck. A: Overall survival in surgery and no surgery group (*P* < 0.001). B: Cancer-specific survival in surgery and no surgery group (*P* < 0.001).

We identified 1443 patients (age of diagnosis >18 years) diagnosed with adenocarcinoma of pancreatic neck from 1998 to 2015 in the SEER data, of which 181 (12.5%) received surgical resection, and 1262 (87.5%) did not receive surgical resection. Table 1 shows the differences in clinicopathologic features in surgically resected and non-surgically managed patients. The majority of patients underwent surgical resection were female gender, young patients, lower grade, and smaller than 2cm of tumor size. Surgical resection was also more likely to be performed in patients with early stage. Receipt of adjuvant therapy (radiotherapy and/or chemotherapy) were more prevalent in patients underwent surgical resection. There was no significant difference in different ethnic populations. The median OS and CSS times were 19 and 21 months in patients underwent surgical resection and 5 and 6 months in non-surgically managed patients. The 1-, 3-, and 5- year OS rates were 66.4%, 23.6%, and 16.4% for surgically resected patients, and 24.9%, 3.0%, and 1.3% for non- surgically resected patients (*P* < 0.001; Fig 1A). In addition, the 1-, 3-, and 5- year CSS rates were 69.7%, 27.1%, and 18.7% for surgically resected patients, and 24.8%, 3.6%, and 1.7% for non- surgically resected patients (*P* < 0.001; Fig 1B).

**Table 1:** Characteristics of patients with adenocarcinoma of pancreatic neck.

	Surgery (%) (n = 181)	No surgery (%) (n = 1262)	*P*
Gender			0.02
Male	68 (37.6)	626 (49.6)	
Female	113 (62.4)	636 (50.4)	
Age (years, mean ±SD)	66.6 ± 11.8	69.4±11.5	0.008
Race			0.054
White	141 (77.9)	1013 (80.3)	
Black	15 (8.3)	140 (11.1)	
Other	25 (13.8)	109 (8.6)	
Tumor size (cm)			<0.001
≤2	46 (27.1)	119 (10.9)	
>2	124 (72.9)	969 (89.1)	
Unknown	11	174	
T stage			<0.001
T1	20 (12.6)	47 (4.8)	
T2	24 (15.2)	209 (21.5)	
T3	100 (63.3)	317 (32.6)	
T4	14 (8.9)	399 (41.1)	
Unknown	23	290	
N stage			0.001
N0	89 (51.1)	654 (64.8)	
N1	85 (48.9)	355 (35.2)	
Unknown	7	253	
M stage			<0.001
M0	166 (93.3)	557 (46.5)	
M1	12 (6.7)	642 (53.5)	
Unknown	3	63	
AJCC stage			<0.001
I	35 (21.1)	77 (6.7)	
II	107 (64.5)	193 (16.9)	
III	12 (7.2)	231 (20.2)	
IV	12 (7.2)	642 (56.2)	
Unknown	15	119	
Grade			<0.001
Well differentiated	14 (9.1)	39 (13.2)	
Moderately differentiated	92 (59.7)	118 (39.9)	
Poorly differentiated	48 (31.2)	131 (44.2)	
Undifferentiated	0 (0)	8 (2.7)	
Unknown	27	966	
Radiotherapy			<0.001
Yes	66 (37.1)	248 (19.8)	
No	112 (62.9)	1003 (80.2)	
Unknown	3	11	
Chemotherapy			0.032
Yes	116 (64.1)	702 (55.6)	
No	65 (35.9)	560 (44.4)	

### Analysis of patients with surgical resection

From the original cohort of patients with surgical resection, a total of 21 patients were excluded because the extent of surgery was unclear (n = 13), or there was metastatic disease (n = 12). This resulted in a following study cohort of 160 patients. Among them, 30 (18.8%) patients underwent DP, 105 (65.6%) patients underwent PD, and 25 (15.6%) patients underwent TP. The characteristics in patients with different types of surgery are shown in Table 2. There were no significant differences in gender, race, tumor size, T stage, AJCC stage, grade, radiotherapy, and chemotherapy between patients underwent DP, PD, or TP. Patients underwent DP were older than these underwent TP (70.5±10.7 vs. 62.2±14.1, *P* = 0.027). However, no other significant differences had been observed between DP group and PD group (*P* = 0.125), or between PD group and TP group (*P* = 0.151). Patients underwent TP had more N1 stage cancers compared to these underwent DP (68.0% vs. 34.5%, *P* = 0.014). No significant N stage differences had been found between DP group and PD group (*P* = 0.164), or between PD group and TP group (*P* = 0.088).

**Table 2:** Comparison of characteristics in patients with different types of surgery.

	Type of surgery			*P*		
	DP (%) (n = 30)	PD (%) (n = 105)	TP (%) (n = 25)	DPvs.PD	DPvs.TP	PDvs.TP
Gender						
Male	8(26.7)	41(39.0)	7(28.0)	0.214	0.912	0.304
Female	22(73.3)	64(61.0)	18(72.0)			
Age (years, mean ±SD)	70.5±10.7	66.8±11.4	62.2±14.1	0.125	0.027	0.151
Race						
White	24 (80.0)	81 (77.1)	19 (76.0)	0.931	0.938	0.974
Black	2 (6.7)	9 (8.6)	2 (8.0)			
Other	4 (13.3)	15 (14.3)	4 (16.0)			
Tumor size (cm)						
≤2	7 (25.0)	30 (29.4)	7 (28.0)	0.814	0.805	1.000
>2	21 (75.0)	72 (70.6)	18 (72.0)			
Unknown	2	3	0			
T stage						
T1+T2	8 (27.6)	27 (28.7)	7 (31.8)	0.906	0.743	0.774
T3+T4	21 (72.4)	67 (71.3)	15 (68.2)			
Unknown	1	11	3			
N stage						
N0	19 (65.5)	53 (51.0)	8 (32.0)	0.164	0.014	0.088
N1	10 (34.5)	51 (49.0)	17 (68.0)			
Unknown	1	1	0			
AJCC stage						
I	8 (27.6)	23 (23.7)	3 (13.0)	0.728	0.427	0.620
II	20 (69.0)	67 (69.1)	18 (78.3)			
III	1 (3.4)	7 (7.2)	2 (8.7)			
Unknown	1	8	2			
Grade						
Well differentiated	2 (7.2)	11 (12.3)	1 (4.5)	0.558	0.362	0.323
Moderately differentiated	20 (71.4)	54 (60.7)	12 (54.5)			
Poorly differentiated	6 (21.4)	24 (27.0)	9 (41.0)			
Unknown	2	16	3			
Radiotherapy						
Yes	11 (36.7)	42 (40.4)	10 (41.7)	0.714	0.708	0.908
No	19 (63.3)	62 (59.6)	14 (58.3)			
Unknown	0	1	1			
Chemotherapy						
Yes	20 (66.7)	70 (66.7)	14 (56.0)	1.000	0.418	0.316
No	10 (33.3)	35 (33.3)	11 (44.0)			

### Survival of patients with surgical resection

**Fig 2:**
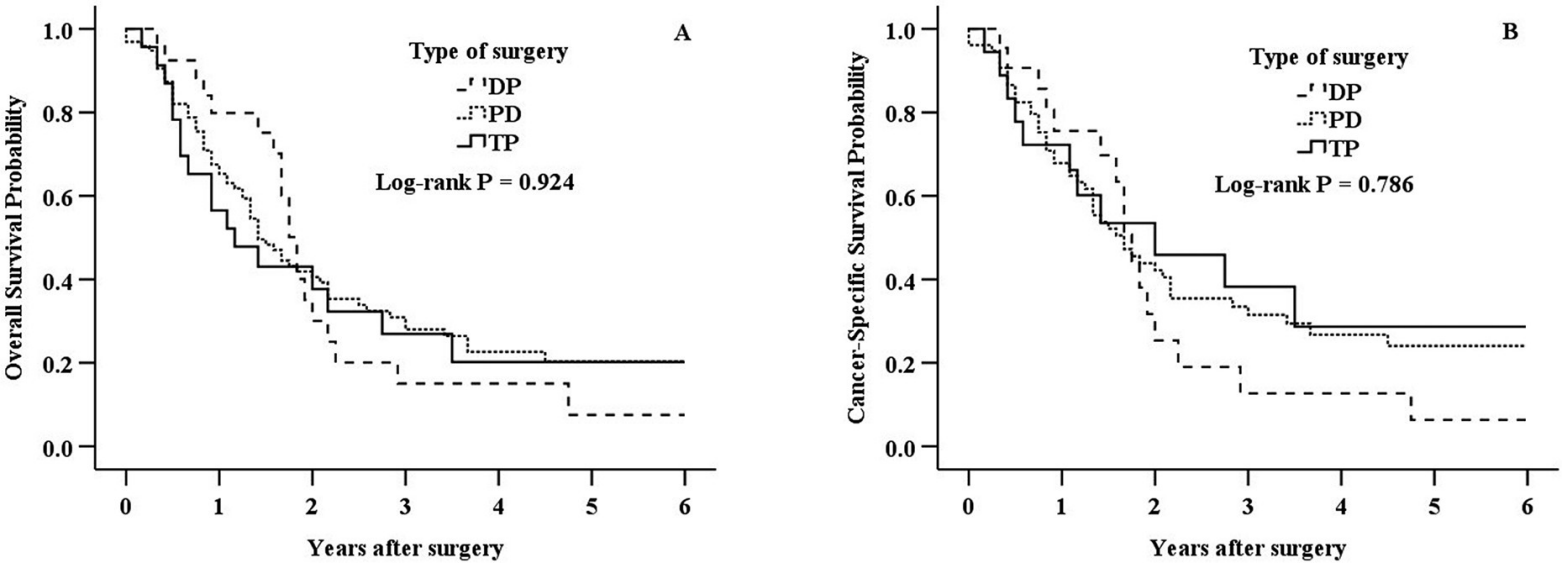
Kaplan-Meier survival analysis of DP, PD and TP patients with adenocarcinoma of pancreatic neck. A: Overall survival in DP, PD, and TP group (*P =* 0.924). B: Cancer-specific survival in DP, PD, and TP group (*P* = 0.786).

**Fig 3:**
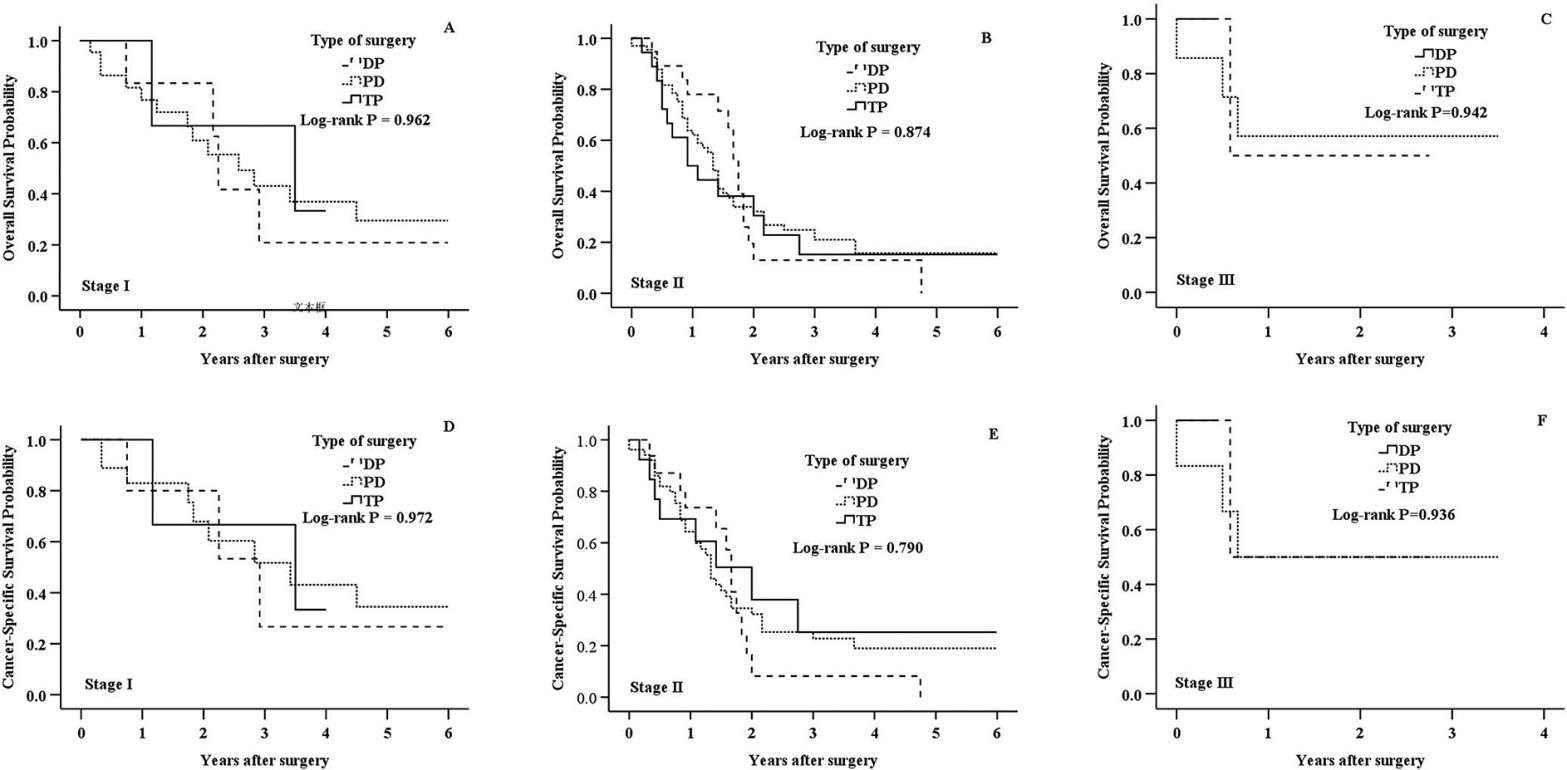
Kaplan-Meier survival analysis of DP, PD and TP patients with adenocarcinoma of pancreatic neck in different AJCC stages. A: Overall survival for stage I in DP, PD, and TP group (*P* = 0.962). B: Overall survival for stage II in DP, PD, and TP group (*P* = 0.874). C: Overall survival for stage III in DP, PD, and TP group (*P* = 0.942). D: Cancer-specific survival for stage I in DP, PD, and TP group (*P* = 0.972). E: Cancer-specific survival for stage II in DP, PD, and TP group (*P* = 0.790). F: Cancer-specific survival for stage III in DP, PD, and TP group (*P* = 0.936).

The median follow-up period was 16 months (range, 0–188 months). Kaplan-Meier survival curves for different types of surgery were shown in Figs 2 and 3. Overall, as shown in Fig 2, all three types of surgery demonstrated similar OS (*P* = 0.924) and CSS (*P* = 0.786). The similarity in survival persisted in univariate Cox regression analysis (Table 3 and Table 4). The multivariate Cox regression model showed that types of surgery were not associated with OS (DP, HR 0.773, 95% CI 0.354–1.685, *P* = 0.517; PD, HR 0.793, 95% CI 0.423–1.484, *P* = 0.468; reference: TP; Table 3) and CSS (DP, HR 1.055, 95% CI 0.419–2.661, *P* = 0.909; PD, HR 0.844, 95% CI 0.386–1.846, *P* = 0.671; reference: TP; Table 4). Comparing the types of surgery by AJCC stage, there was no significantly association with either OS or CSS in any subgroup of AJCC stage (Fig 3).

**Table 3:** Univariate and multivariate analysis of overall survival in patients with surgery.

	Univariate			Multivariate		
	HR	95%CI	*P*	HR	95%CI	*P*
Gender						
Male	1					
Female	0.785	0.534–1.152	0.216	0.767	0.471–1.248	0.285
Age (years)						
≤70	1					
>70	1.876	1.200–2.932	0.006	1.370	0.813–2.310	0.238
Race						
White	0.973	0.562–1.685	0.922	1.256	0.648–2.434	0.499
Black	0.847	0.369–1.943	0.695	2.347	0.831–6.630	0.107
Other	1					
Tumor size (cm)						
≤2	0.805	0.523–1.238	0.323	1.324	0.781–2.244	0.297
>2	1					
AJCC stage						
I	1					
II	1.815	1.105–2.980	0.019	2.266	1.164–4.411	0.016
III	1.140	0.388–3.350	0.811	6.840	1.693–27.631	0.007
Grade						
Well differentiated	1					
Moderately differentiated	2.711	1.089–6.747	0.032	4.063	1.174–14.062	0.027
Poorly differentiated	3.361	1.305–8.655	0.012	4.269	1.173–15.537	0.028
Radiotherapy						
Yes	1					
No	1.443	0.982–2.119	0.062	1.571	0.873–2.829	0.132
Chemotherapy						
Yes	1					
No	1.324	0.902–1.941	0.151	1.475	0.828–2.627	0.187
Type of surgery						
DP	0.855	0.456–1.605	0.626	0.773	0.354–1.685	0.517
PD	0.890	0.537–1.477	0.635	0.793	0.423–1.484	0.468
TP	1					

**Table 4:** Univariate and multivariate analysis of cancer-specific survival in patients with surgery.

	Univariate			Multivariate		
	HR	95%CI	*P*	HR	95%CI	*P*
Gender						
Male	1					
Female	0.772	0.495–1.204	0.254	0.992	0.549–1.795	0.980
Age (years)						
≤70	1					
>70	1.770	1.075–2.914	0.025	1.113	0.592–2.094	0.739
Race						
White	0.937	0.514–1.709	0.833	1.168	0.575–2.370	0.668
Black	0.729	0.289–1.835	0.502	2.350	0.739–7.466	0.148
Other	1					
Tumor size (cm)						
≤2	1					
>2	1.332	0.803–2.212	0.267	1.576	0.833–2.981	0.162
AJCC stage						
I	1					
II	2.040	1.132–3.675	0.018	2.777	1.199–6.431	0.017
III	1.481	0.485–4.516	0.490	14.314	3.046–67.277	0.001
Grade						
Well differentiated	1					
Moderately differentiated	3.320	1.195–9.226	0.021	6.117	1.316–28.421	0.021
Poorly differentiated	3.497	1.192–10.256	0.023	5.983	1.241–28.847	0.026
Radiotherapy						
Yes	1					
No	1.610	1.016–2.554	0.043	2.386	1.175–4.844	0.016
Chemotherapy						
Yes	1					
No	1.220	0.774–1.923	0.391	1.087	0.553–2.139	0.808
Type of surgery						
DP	1.345	0.634–2.852	0.440	1.055	0.419–2.661	0.909
PD	1.068	0.570–1.999	0.838	0.844	0.386–1.846	0.671
TP	1					

In the univariate Cox regression analysis, age older than 60 years (HR 1.876, 95% CI 1.200–2.932, *P* = 0.006; Table 3), AJCC stage II (HR 1.815, 95%CI 1.105–2.980, *P* = 0.019; Table 3), higher tumor grade (Moderately differentiated, HR 2.711, 95% CI 1.089–6.747, *P* = 0.032; Poorly differentiated, HR 3.361, 95% CI 1.305–8.655, *P* = 0.012; reference: Well differentiated; Table 3) were predictors of poor OS. In addition to the above factors, no radiotherapy was another factor associated with poor CSS (HR 1.610, 95% CI 1.016–2.554, *P* = 0.043; Table 4). In the multivariate Cox regression analysis, most of these factors remained independent prognostic factors, with the exception of age (*P* = 0.238 for OS; *P* = 0.739 for CSS; Tables 3 and 4). Additionally, AJCC stage III was a significant independent prognostic factor for OS (HR 6.840, 95% CI 1.693–27.631, *P* = 0.007; Table 3) and CSS (HR 14.314, 95% CI 3.046–67.277, *P* = 0.001; Table 3).

## Discussion

The surgical management remains the only curative treatment for pancreatic adenocarcinoma. PD, DP, and TP are regarded as standard procedures in the treatment of pancreatic duct adenocarcinoma and should be performed according to tumor location.[7] Much debate has focused on the selection of optimal surgical procedures.[3,8] In this population-based study, we analysed the treatment practices for patients with adenocarcinoma of pancreatic neck and assessed the prognostic factors. This study showed that only 12.5% patients had undergone surgical resection with better OS and CSS compared to those with no surgery. The Kaplan-Meier analysis demonstrated that the OS and CSS in the DP, PD and TP groups did not differ significantly. Even if in subgroups of AJCC stage, similar results were found. Considering the factors on survival, the multivariate analysis showed that types of surgery were not associated with prognosis. On the contrary, higher AJCC stage and grade were independent prognostic factors for poor OS and CSS. In addition, no radiotherapy was another factor associated with poor CSS. Some population-based data have evaluated the association between surgical procedures and prognosis based on tumor location, with the exception of pancreatic neck. Nathan el at.[9] found, by using SEER database, that long-term survival was similar following TP versus partial pancreatectomy (e.g. PD and DP) for pancreatic adenocarcinoma in different tumor location (HR 1.06, P = 0.49 for head; HR 0.84, P = 0.51 for body/tail; HR 1.06, P = 0.79 for unspecified locations). Also using SEER database, Govindarajan et al.[10] found that there had been no significant difference in survival between TP, PD and pylorus-preserving pancreaticoduodenectomy for cancer of pancreatic head.

Pancreatic neck locates in a narrow section between pancreatic head and body, adjoining to PV, GDA, and CHA. The rates of PV and/or superior mesenteric vein invasion were more frequent in patients with pancreatic neck cancer than those with pancreatic head and body/tail cancers.[4] In case of the anterior surfaces of these veins were involved, it is difficult to establish a tunnel behind the pancreatic neck, and these tumors are commonly considered unresectable, when in fact it is safely resectable by experienced pancreatic surgeons.[11] Furthermore, due to the general nihilistic attitude that still exists in many parts of the United States with respect to this disease, 38.2% early stage pancreatic cancer patients without any identifiable contraindications were not offered surgery.[12] These findings may help explain why only 12.5% patients have received surgical resection in our study, and less resectable than in the pancreatic head and body/tail (29.9% and 16.1%, respectively), reported by Artinyan et al.[13] using the SEER database. To deal with these difficult tumors, Strasberg et al.[11] performed an innovative surgical technique named as “Whipple at the splenic artery”, in which the point where the splenic artery comes onto the superior border of the pancreas was chosen as the site of transection. In 2013, another evolutive procedure named as “Whipple at the inferior mesenteric vein” was described by our center, in which the point where the right portion of the inferior mesenteric vein enters into the inferior border of the pancreas was chosen as the site of transaction.[14] This procedure had comparable postoperative morbidity with standard PD with vein resection procedure.[15]These two techniques belong to the category of proximal subtotal pancreatectomy. Park et al.[16] described extended DP in patients with pancreatic neck cancer accompanied by distal pancreatic atrophy, preserving only the uncinate process of the pancreas. The aim of a surgical technique is to achieve radical tumor removal, which is a precondition for good survival in patients undergoing surgery for pancreatic adenocarcinoma.[7] For this purpose, TP is an alternative surgical procedure in high-volume pancreatic centers.[3]

Most published reports on TP have been focused on comparison with PD.[8,17–21] The role of TP has historically been limited due to higher perioperative morbidity and mortality rates.[8] Additionally, several metabolic problems leading to a poor quality of life (QOL) are co-related with the apancreatic state.[22] Recently, with advances in surgical techniques and pre-, peri- and postoperative care, TP has been increasingly indicated. There are several studies that have shown that major morbidity and mortality of TP has been almost equivalent to that of the PD performed.[20,21] The comparison of patients who underwent TP and PD showed no statistically significant differences in overall QOL.[23] However, the long-term survival was not significantly improved.[9,18,20,21] These findings are consistent with our study, in which clinicopathological features were comparable between TP and PD group. Therefore, although TP can drastically reduce the lesion, the improvement in patient prognosis was not remarkable. The reason why TP is not superior to PD may be because of the long-term survival of patients undergoing TP is dependent on the biology of the underlying cancer.[3] As we found in this study, AJCC stage and tumor grade were independent prognostic factors. In cases when arterial reconstructions are undertaken, the complete removal of the pancreas makes the procedure safer by eliminating completely the problem of pancreas fistula and it’s potentially fatal effect on the arterial anastomosis.[24,25] In cases when isolated pancreatic neck margin is positive after PD, conversion from PD to TP to achieve an R0 resection in patients with pancreatic adenocarcinoma is associated with a better survival.[17] However, Desaki et al.[26] performed a proximal subtotal pancreatectomy with splenic artery and vein resection, so-called pancreaticoduodenectomy with splenic artery resection. They found 88.9% patients could obtain negative pancreatic margin and avoid TP. Thereby, TP should be considered in carefully selective cases for treatment of adenocarcinoma of pancreatic neck if it allows complete clearance.

To our knowledge, study comparing TP and DP is limited. The reason for this observation might be (1) DP is a conventional procedure on treatment of tumor in pancreatic body/tail. (2) TP may not have been a clinically or oncologically appropriate alternative in these cases.[22] Nathan et al.[9] demonstrated that one-month mortality and long-term survival were similar between TP and DP for pancreatic adenocarcinoma in pancreatic body/tail. Hank et al.[27] found an inconsistent result that DP was associated with a decreased risk of shorter survival compared with TP for adenocarcinomas of the pancreatic body and tail. In our study, OS and CSS were similar between TP and DP group for adenocarcinoma of pancreatic neck, despite the fact that the percentages of nodal metastasis (N1) were higher in TP.

Moreover, no data is available about the survival rate of patients when compared PD with DP in same location of pancreatic cancer. Ruess et al.[28] reported that patients with resectable pancreatic adenocarcinoma located in the body and tail of the pancreas (undergoing DP) display a similar morbidity, mortality, and 5-year survival rates when compared to patients with resectable tumors located in the pancreatic head (undergoing PD). However, early stage of pancreatic cancer in the pancreatic body and tail may be associated with superior survival compared with those in the pancreatic head.[29] This finding is most likely due to pancreatic body and tail cancer presents a less malignant phenotype associated with deregulation of miR-501-3p compared with pancreatic head cancer, at resectable early stage.[29] In our study, there was no statistic difference of clinicopathological features between PD and DP group. OS and CSS were comparable between the two groups for adenocarcinoma of pancreatic neck.

Due to its anatomic location, pancreatic neck cancer may frequently invade the major arteries, such as the CHA, the celiac axis, and/or the superior mesenteric artery, which are generally considered as borderline resectable or unresectable disease.[4] Although resections can often technically be performed in these cases, R0 resection is difficult to perform in case of central involvement of these arteries.[30,31] Neoadjuvant therapy may lead to successful R0 resection and promote long-term survival.[32] For patients with resectable pancreatic cancer, upfront resection followed by adjuvant chemotherapy is the standard approach.[33] The survival data from randomized trials designed to investigate the efficacy of adjuvant therapy after upfront resection highlight the considerable advances that have been made.[34–36] Although chemotherapy has been shown to consistently improve outcomes, the data regarding radiation therapy is conflicting.[37,38] However, the use of neoadjuvant radiation in borderline resectable and locally advanced disease is much more accepted than its use in resectable tumor patients.[39] Using the SEER database, Sajjad et al.[40] demonstrated a clear survival benefit in patients with locally advanced pancreatic cancer who received radiation therapy. This is consistent with the result of our study that CCS of patients underwent surgery is influenced by treatment of radiation. However, receive of chemotherapy is not associated with OS and CSS. The reason for this observation might be we could not get the data on chemotherapy regimens and the actual duration/number of successful cycles received by each patient from the SEER database.

The SEER database includes a large nation-wide cohort of patients with pancreatic cancer in the United States, whereas there are several limitations in our study. Firstly, as with any retrospective study evaluating a surgical modality, selection bias may have affected the allocation of DP, PD and TP. In addition, it was unclear that the selection of surgical procedure was based on preoperative imaging results or intraoperative exploration. Secondly, The SEER database does not provide following information: margin status, detailed regimens and the timing of chemoradiotherapy which can have an effect on our primary outcomes of OS and CSS. In addition, unavailability of variables such as morbidity, which is an important outcome when studying variations in surgical procedures. Thirdly, the definition of the anatomic location of the pancreatic neck remains somewhat obscure,[4] and may not be consistently used by all abstractors providing information to SEER. Finally, the limited size of pancreatic neck adenocarcinoma patient, especially in subgroup analysis (TP and DP) may reduce statistical power.

In conclusion, this study suggests that patients with adenocarcinoma of pancreatic neck who are treated with surgical resection have a survival benefit. There is no evidence to demonstrate that different types of surgery have an impact on prognosis. Considering the underlying side effects of TP, it cannot be considered the standard of care for the surgical treatment of patients with adenocarcinoma of pancreatic neck. Due to the limitations caused by the lack of key variables in the SEER database, further prospective large-cohort study regarding pancreatic neck cancer with consistent definition is needed.
